# Supplementary material for: Meso level influences on long term condition self-management: stakeholder accounts of commonalities and differences across six European countries
Source: BMC Public Health. 2015 Jul 8;15:622. doi: 10.1186/s12889-015-1957-1 (PMC4495781; doi:10.1186/s12889-015-1957-1)
Supplement: Additional file 1: — Appendix: Country specific policy related to the management of long-term conditions. [file 12889_2015_1957_MOESM1_ESM.docx]

Meso level influences on long term condition self-management: stakeholder accounts of commonalities and differences across six European countries

Anne Rogers1 a.e.rogers@soton.ac.uk
Corresponding Author

Ivaylo Vassilev1, i.i.vassilev@soton.ac.uk

Maria J. Jesús Pumar2 mnavarta@unav.es

Elka Todorova 3, elka.todorova@me.com

Mari Carmen Portillo 2, mportillo@unav.es

Christina Foss 4 i.c.foss@medisin.uio.no

Jan Koetsenruijter5, Jan.Koetsenruijter@radboudumc.nl

Nikoleta Ratsika6, ratsika@staff.teicrete.gr

Manuel Serrano7, manuelserranogil@gmail.com

Ingrid A. Ruud Knutsen4, i.a.r.knutsen@medisin.uio.no

Michel Wensing5, Michel.Wensing@radboudumc.nl

Poli Roukova3 proukova@bas.bg

Evridiki Patelarou6, evridiki.patelarou@kcl.ac.uk

Anne Kennedy1, a.kennedy@soton.ac.uk

Christos Lionis 6 lionis@galinos.soc.med.uoc.gr

1NIHR CLAHRC Wessex, Faculty of Health Sciences, University of Southampton Highfield Campus University Road Southampton SO17 1BJ UK

2School of Nursing. University of Navarra, Spain

3Department of Sociology, University of National and World Economy, & Department of Economic and Social Geography Bulgarian Academy of Sciences Sofia Bulgaria

4Dept. of Nursing Sciences, Institute of Health and Society, University of Oslo, Norway

5Scientific Institute for Quality in Healthcare, Radboud University Medical Centre, Netherlands

6Clinic of Social and Family Medicine, School of Medicine, University of Crete, Greece
Funducaion Educacion, Salud Y Sociedad, Murcia, Spain

Abstract

Background: European countries are increasingly adopting systems of self –care support for long term conditions which focus on enhancing individual, competencies, skills, behaviour and lifestyle changes. To date the focus of policy for engendering greater self- management in the population has been focused in the main on the actions and motivations of individuals. Less attention has been paid to how the broader influences relevant to SMS policy and practice such as those related to food production, distribution and consumption and the structural aspects and economics relating to physical exercise and governance of health care delivery systems might be implicated in the populations ability to self- manage rarely. This study aimed to identify key informants operating with knowledge of both policy and practice related to SMS and how these influences are seen to impact on the self-management support environment for diabetes type 2.

Methods: 90 semi-structured interviews with key stakeholder informants in Bulgaria, Spain, Greece, Norway, Netherlands and UK. Interviews were transcribed and analysed using thematic and textual analysis.

Results: Stakeholders in the six countries identified a range of influences which shaped diabetes self-management (SM). The infrastructure and culture for supporting self-management practice is viewed as driven by political decision-makers, the socio-economic and policy environment, and the ethos and delivery of chronic illness management in health care systems. Three key themes emerged during the analysis of data. These were 1) social environmental influences on diabetes self-management 2) reluctance or inability of policy makers to regulate processes and environments related to chronic illness management 3) the focus of healthcare system governance and gaps in provision of self-management support (SMS). Nuances in the salience and content of these themes between partner countries related to the presence of dedicated prevention policies articulation of self-management policies and behavioural interventions in primary care , drug company involvement and the impact of measures resulting from economic crises, and differences between countries with higher versus lower social welfare support and public spending on shaping illness management.

Conclusions: The results suggest reasons for giving increasing prominence to meso level influences as a means of rebalancing and improving the effectiveness of implementing an agenda for SMS. There is a need to acknowledge the greater economic and policy challenging environment operating in some countries which act as a source of inequality between countries in addressing SMS for chronic illness management.

Key words: Self-management support, Long-term conditions, diabetes Type 2, macro, meso level influences, state capacity, cross-national comparison

Background

Recent theoretical and empirical investigation of SMS point to the implicit impact of wider structural influences on long term condition management and the capacity of people to self – manage. Previous studies have illuminated how the quality of support for long term conditions is influenced by individual social economic status, by residential area deprivation, social and employment status availability of locality social capital and opportunities for social involvement [1,2,3]. Notwithstanding this evidence some facets of the influence of the broader socio- economic and policy environment on capacity to self- manage remain relatively under-explored . Thus, the aim here is to extend knowledge and understanding through research of structural and meso level policy and other influences operating in different socio-cultural settings which are likely to influence capacity to self-manage a long term condition. This research focus is relevant to identifying the modifications at a non-individual level needed to amend current approaches and policy in relation to self care support.

European countries are increasingly adopting systems of long term condition management which include formulations for the delivery of self-management support (SMS) which stress individual motivation, goal setting, problem solving, life-style modification and information provision . For conditions, such as diabetes type 2, attention has been paid to addressing the complexities of adjusting behaviour and practices through multiple behavioral and lifestyle change interventions [4,5]. Less attention has been paid to the contributions that may shape the uptake of SMS made by broader level influences which have been identified as increasingly relevant in an era of increasing economic crisis and uncertainty [1,6]. The latter points to the need to more fully explore the economic and social policies related to food production, distribution and consumption, the structural aspects and economics of the environment relating to exercise and the structure and governance of health care delivery systems at a local and regional level [7]. In relation to type 2 diabetes specifically a recent realist review concluded that “Socio-economic circumstances are relevant to the capacity to self-manage and suggest that any gains and progress will be hard to maintain during economic austerity” [6].The study of influences operating across different European countries are relevant for balancing out the predominant focus of individual strategies in considering future public health and policy initiatives in this area. There is for example recent evidence pointing to the relevance of collective and network contributions of support for chronic illness and for the need to address inequalities in health provision in different European countries [8,9,10,11]. Political science approaches have pointed to the tension in policy making between different interests operating within the welfare- state. The latter is deemed to result in a number of tensions around the configuration of policy. This is particular evident in policies around population health and prevention [12]. In this respect a number of extra individual influences are likely to be relevant to SMS but currently these are poorly conceptualised and articulated at a policy level. What currently remains hidden from view is how the environment of those with long terms conditions is shaped by the interests of incidental political and economic interests and how the environment for self-management is shaped by access to financial and social resources [13,14]. As well as similarities there are likely to be cultural and contextual distinctions regarding broader influences between European countries. Using diabetes type 2 as an orientating condition our aim was to rebalance the focus on the micro individual action focus of policy through

tapping the hidden but relevant, predominantly meso level influences of political-economic, policy, and institutional organisational arrangements, by examining perspectives of key stakeholders.

Self management has been traditionally defined as “the care taken by individuals towards their own health and well-being: it comprises the actions they take to lead a healthy life style, to meet their social, emotional and psychological needs to care for their long term condition and to prevent further illness or accidents”[15]. Self management support (SMS) includes the development of new technologies, information, training, support networks and professional help. Here we focus on the influences which are related to the political and economic context and institutional arrangements shaping health policy and practice for long term conditions. In this regard the focus here is on meso level policy and institutional contexts shaping the SMS environment. This cross-national analysis aims to better understand how and what multi-level factors operate and impact on the SMS environment it is necessary to go beyond the documented evidence to tap more hidden knowledge not easily accessible from more traditional sources.

Methods

This paper builds on work conducted by the Framework 7 EU-WISE project, which seeks to understand the environmental influences on chronic illness management in order to inform and develop future initiatives relevant in peoples’ everyday life across selective settings in Europe (http://eu-wise.com/). Here we report on the findings from key informant interviews conducted in Greece (GR), Spain (ES)[1], Bulgaria (BG), Norway (NO), United Kingdom (UK), and Netherlands (NL). The partner countries represent a range of socio-economic contexts and institutional and policy arrangements that could have an impact on the organisation and experiences of SMS.

Key informant interviews

Table 1: Background of key informant interviews (INSERT HERE)

We conducted a set of semi-structured interviews with key informants involved in or familiar with SMS policy, practice and research (see Table 1).

We approached diverse set of indivdual stakeholders who were likely to be able to offer varied experience and represent diverse interests. Individuals in prominent, policy, industry, opinion forming and academic leadership positions who were likely to be immersed in inside knowledge at the interface between research, policy and practice. Participants were identified through purposive and snow-balling sampling techniques drawn from networked contacts and personal knowledge in each of the partner countries. Media sources (for

[1] Data in Spain was collected in two different parts of the country. The relatively poorer southern region of Murcia and the relatively more afluent northern region of Navarra. These differences were taken into account when presenting the data within th ecomparative context of th estudy.

policy statements) and websites of organisations were accessed to confirm the profile of the person to interview was relevant to different aspects of SMS for diabetes type 2. Each of these individuals was expected to have knowledge, which was not held by others, thus we were not necessarily expecting saturation in the interviews. The number of 15 informants was pragmatically determined, assuming that a diversity of views could be documented in this way.

Potential interviewees were directed to the EU-WISE website and sent an information and consent form explaining the background to the study. Participants were given a list of topic areas we were interested in pursuing, which included:

- *New policies/practices in health systems*
- *Economic/social environment and the "age of austerity"*
- *Academic, clinical, policy thinking about optimal chronic disease and SM*

90 interviews (15 in each country) were conducted with key informants representing academics involved with research in the area of primary care, public health, diabetes type 2 SMS, the private and public sector providers, practitioners, voluntary organisations and policy makers. Respondents were identified using purposive and snowball sampling aiming to tap into a wide range of opinions, interests, and experiences. The interviews lasted between 30 and 90 minutes and were conducted by project team members or by experienced qualitative interviewers who received training about the broad aims of the project and the objectives of the stakeholder interviews. An interview guide was created after an expert group discussion, which included questions for the main thematic areas that were covered during the interviews (Box 1). Some of the topics which were introduced were informed by a realist review of diabetes self- management arrangements in Europe [6]. The topics translated into qutestions from the review included the following: i) the relevance of socio-economic circumstances to the capacity to self-manage and maintain during economic austerity. ii)Success in achieving individual control needs further interpretation within the wider context of a whole systems approach regarding self-care support and chronic illness management. Iii) The success of self-management as a policy solution will be affected by interacting influences at three levels: [a] at micro-level by individuals' dispositions and capabilities; [b] at meso-level by roles, relationships and material conditions within the family in the workplace, school and healthcare organisation; and [c] at macro-level by prevailing economic conditions, cultural norms and expectations, and the underpinning logic of the healthcare system and policy .iv) the need for evidence on broader welfare systems and economies of partner countries along with current initiatives and community services. and austerity policies by the economic crisis.

Box 1: Interview guide (adapted to each partner country)

- What are the key changes, policies, innovations in SMS and diabetes type 2 over the last 10 years? Why have these been the most important ones? What changes have these led to?
- Why do you think policy has changed in the way that it has?
- Who are the most important stakeholders in this area? How have they influenced the agenda around SMS?
- What is the role of drug companies nationally internationally? Do you have a view of current policy around the role of drug companies or how they influence the agenda in this area?
- What is the involvement of private companies in SMS (e.g. through lifestyle programmes and subcontracting of health related local services)?
- How is the broader healthcare system organised to support long term condition management?
- What is the role of health prevention and policy in SMS?
- What are government attitudes to lifestyle and behavioural changes at the level of patients?
- What are the funding and incentives structures in the health system, particularly at the level of public health and primary care?
- What programmes and policies are there for the prevention of long term conditions , guidelines for monitoring, prescribing, and care for diabetes type 2?
- What are the public attitudes to SMS and diabetes type 2?
- What have been the main media constructions of the epidemic of diabetes type 2 and who is at risk?
- How has policy around and towards inequalities had an impact?
- What if any is the impact of the fiscal crises?

Data processing and analysis

Interviews were recorded and transcribed verbatim in Spanish, English, Bulgarian, Greek, Norwegian, and Dutch[2]. Informed consent was obtained prior to interviewing. The transcripts were analysed using thematic analysis [16]. Initial, line-by-line coding of the findings of each partner countries transcripts were organised into 'free codes' in related areas to construct 'descriptive' themes and the development of 'analytical' themes. The latter process incorporated working out elements of the salient phenomena from a first reading of the transcripts and analysing these further (analytic resolution) in stages first within each partner country and then through data analysis clinics across countries. We used a sensitising schema which included a range of influencing factors at a macro level which where there were suggestions of influence at the- meso level and at a theoretical level seen as potentially impactful at the level of the micro (i.e. individual action). Macro factors referrred to state, global, regional organisational and cultural influences, whilst meso ones refer to local organisational arrangements, guidelines. We used this framing of SMS to illuminate implied connections with extra- individual influences and subtle interrelationships that might exist and also illuminate cross cultural similarities and differences.

[2] Some of the Dutch interviews were not recorded. Non-recorded interviews were analyzed on the basis of detailed typed summaries that we made immediately after each interview.

The thematic framework emerged from analysis of a preliminary set of interviews from each partner which were collated and shared with other partners to arrive at a consistent set of themes across settings in six countries (Crete, Bulgaria, England, Netherlands, Spain, Norway). Each participant country undertook reading and re-reading of transcripts and field notes which forming the bases of thematic and textual analysis arriving at a set of key themes and re-current sub-themes (see Table 2). Relevant quotes were translated into English and discussed with all partner countries involved[i]. We undertook two comparative data analysis clinics with each participating partner. This was followed by additional discussions with individual partner countries. Each partner's initial coding was subjected to an adapted comparative method to identify convergent and divergent themes across topics. The process was inductive and flexible. Consensus of the meaning of key topics was obtained through the cross cultural analysis undertaken in the data clinics with the researchers from each country working towards shared meanings.

Ethics

Ethical approval was granted by the relevant ethics committees in each partner country [ii]. Bulgaria NCPHA (National Center for Public Health and Analysis).Netherlands CMO region Arnhem Nijmegen, number 2013/098; Spain the University of Navarra. Reference number: 014-2013University of Southampton Ethics and Research Governance Online, (ref. 4340) Norway REK 2012/593b and OUS-HF 2013/2922. Bio-ethical Committee and the Administration Council of the Regional Academic Hospital (PAGNI) of Heraklion Crete (*Date: 27/06-12-2012, No. 1087- 644/27/31-7-2012, Decision 974/26/20-11-2012)* All participants gave informed consent for taking part in the study and their contributions are anonymised in the presentation of the data.

Findings

From the data analysis undertaken three broad themes emerged: 1) social environmental influences on diabetes SM (stigma, inequalities and food) 2) reluctance or inability of policy makers to regulate processes and environments related to chronic illness management, 3) biomedical focus and gaps in provision of SMS in the healthcare system (see Table 2).

Table 2: Key themes and sub-themes (INSERT HERE)

Through this process of analysis we looked at the range of factors across the countries and presented data to show a clustering around key themes (see table 2).

TABLE 3: Key similarities and differences between partner countries (insert here)

Social environmental influences on diabetes self-management: inequalities, stigma and food

The most prominent social environmental influences identified as impacting on capacity for diabetes self- management were social inequalities, stigma, and the presence of a diabetogenic food environment. Social inequalities were identified as having a long term impact on peoples' resources to self-manage. Respondents in the UK, NL and NO discussed unequal access to resources including the impact of social inequalities on prenatal predispositions, access to resources within urban planning policies (e.g. the location of food outlets, transport and exercise opportunities, diabetic 'friendly' diets, housing and health education).

The impact of austerity and economic circumstances were forefronted in the more economically deprived countries of the partner countries (BG, GR) as producing a fateful impact on access to diet and healthy lifestyle options.

> *We shouldn't forget that a large part of the patients are elderly people and they have no financial means for internet access – with an average of 300 BGN they have to pay for overheads, utilities, stick to a healthy diet, buy the necessary medicines (BG, 12).*

> *For the last few years in Greece during the economic crisis, everyone is left to their own fate. In other words, social disparities are exacerbated to the point where access to expensive therapies or to certain other therapies is very difficult.......; it's the fact that there's an increase of the uninsured, and inequalities are dramatically increasing (GR, 1).*

Growing public awareness about the presence and causes of diabetes was viewed as a trend across all partner countries. Some argued that knowledge about its causes and management failed to negate the promulgation of stigma towards those at risk of or living with diabetes type 2. Negative attributes included over- emphasising individual responsibility for the development of diabetes through poor lifestyle choices leading to obesity. The media was identified in playing a role here.

> *There are certain messages that might unnecessarily alarm the population, or they might broadcast the wrong messages that damage us all. I think we should be much more responsible…. we use the media to defend our personal opinions, our working conditions, etc. and all this does nothing but distort, s (SP, 9).*

> *I think that there are very negative stereo types about fat people ... last year's national diabetes week The Times ran some articles with pictures and they were just grossly horrible pictures of very, very fat people and in a sense most people with diabetes are a bit overweight, I mean they are overweight but they're not grossly overweight. … I don't think that the media have a really helpful, positive way about helping us all…, I think they rather like to peepshow (UK, 5).*

The quality of the food environment was most prominent in interviews in UK and NO as a factor shaping diabetes SM. The point was made that a diabetogenic environment and production of unhealthy food were implicitly promoted through the availability of subsidies. Further down the supply chain foods containing high sugar and fat content were offered at

low prices and located in food outlets in a way which made it likely that they would be chosen by consumers.

> *…. Why should a green apple be more expensive than a Mars bar? Put tax on the Mars bar, tax the Mars bar, subsidise fresh fruit, you have an exchange. […] that's how you change behaviour…. Why don't you subsidise the shops not to have any sweets and chocolates and their exits or rather put bits of fruitThe financial incentives are absolutely critical around food policy, you know we can focus on in labelling but people are habitual in the food they eat (UK, 8).*

Inability of policy to regulate processes and environments relevant to chronic illness management

The inability of policy makers to regulate environments effectively was attributed to the complexity of dealing with the responsible influences. Reference was made to the diminishing financial and regulatory capacity of States to act to growing population needs, the influence of entrenched vested interests of food and pharmaceutical companies and users of transport infrastructure. The lack of state capacity to intervene in shaping diabetes environments was discussed by informants in all partner countries. However, there was a clear difference in emphasis with respondents in the countries with higher welfare and public spending identifying political beliefs, ideology, and willingness of policy makers as the main factors shaping the self-management support environment. In countries with few welfare state resources for these activities (Southern and East European contexts limited state capacity and the need to prioritise that were cited as being important. For example, in relation to food regulation our UK respondents argued that:

> *the way our current Government operate is very hands off and very kind of personal responsibility… I do believe that we need to not just work with the major food organisations …that to actually put very, very firm pressure on them from the centre to actually change their behaviours. Now that's not something that one would expect a right leaning Government to do.[…] We are going to have to make it easier, and again in terms of behaviour change to me the theory is just about making things easier for people.. (UK, 11)*

In contrast BG and GR respondents referred to the lack of public health policies to illustrate the point.

> *The system tolerates turning patients into clients of the system, not into cured healthy individuals. Prophylaxis is not tolerated and the health fund pays only after a patient has become chronically ill (BG, 8).*

The consequences of the lack of state capacity and state withdrawal from the regulation of Chronic Illness Management (CIM) differed between those with more welfare support and public spending and those with less. In UK, NO, and NL there is a tendency towards shifting responsibility for CIM downwards to the local level and the integration of services closer to users.

… In the end we would like to achieve a connection between the primary care and the local activities. So that a GP knows that he could send a patient to, for example, sport and exercise program. GPs however find it hard to choose the right program because of the large amount of options. And that's what we would like to achieve, to create a vital neighbourhood where the care is efficient; where professionals know which care is offered and can cooperate (NL, 15).

The process of localisation and integration of services drew comments relating to under-resourcing. Local stakeholders felt that responsibility is devolved and solutions expected to "naturally" emerge in the absence of adequate support, guidance, and funding.

. Well they are disappointed because they want to work more on health promotion and prevention than they can. But they have to prioritise the seriously ill first. So the municipalities are disappointed that finances are not following the reform (NO, 2).

Yes obviously we're extremely closely linked to the local authority, they own these buildings, they pay us to operate them, if they are feeling a squeeze, then we will feel a squeeze …, I anticipate that there will be some impact on facilities…, and that will therefore impact on us because it will be our staff, .., it kind of flows down from local authority… (UK, 7).

These processes were not identified by respondents in BG, GR or ES despite the latter having a highly decentralised administrative structure. However, respondent accounts across partner countries pointed to a sense of a subtle duplicity evident in policies that promoted exercise over the need to take action over food for example by providing structural solutions to being able to exercise.

Bio-medical tendencies and incentives in primary care

A strong biomedical focus in the orientation of management from within the healthcare service was evident in partner countries, but especially in BG, GR, and ES.

We have now a system oriented towards medical services, so now we need to reorganize ourselves and shift to a more open system, more towards the community, towards the patients,... let the patient have their support from community nurses and hospital nurses. Then all this needs a different model and the organization of resources in a different way (SP, 12).

Most respondents indicated that there was a gap between awareness of good SMS practice, the development of policy initiatives and their implementation through the healthcare system. There were contrasts between wealthy northern European countries and poorer south and EE countries of translation into policy and practice. The main challenges identified by respondents in NL, UK, and NO referred to implementation. In some cases a implementation failure was attributed to unawareness among policy makers of the (lack of)

impact of existing SMS policies or the absence of a sufficiently strong political commitment to drive through initiatives.

> *Well to be honest, there have been no dramatic changes at all. It hasn't, well it was the diabetes strategy – the diabetes strategy I worked with for some years. It was many nice words, but it has not materialized in changes. There have been many good plans, but there are no political or economic consequences of them (NO, 4).*

SMS policy initiatives may fail to impact on practice even where steps are taken towards implementation because of the absence of whole system change. I SMS requires the development and implementation of dedicated tools and measurement some of which are under development which compete with more established bio-medical tools and measures already in use. Respondents referred to the need to clarify priorities.

> *… a little more clarity if possible around incentives I'm uncertain about, and some real consistency about metrics, you know the metrics really don't line up at the moment, so what are we trying to achieve, how are we going to measure what we are trying to achieve, bang, bang, bang. We haven't got that consistency (UK, 11)*

> *What I mean is that it is a chaos of information out there, and my opinion is that we as official actors should promote the normative guidelines. This is the way we treat diabetes in Norway today. So I think we need better clinical guidelines that might be adapted to primary health care. Since diabetes treatment changes quickly – for a GP to be updated and who just have a few diabetes patients in his 1500 patient list, you need good and accessible information (NO, 4).*

Priorities of health care organisations might be incompatible or contradictory to SMS. For example non-incentivised items tend to be downplayed through a prioritisation of the bio-medical focus of incentivised work (e.g. via the Quality and Outcomes Framework (QOF) in the UK [17].

The lack of specification as to who is responsible for delivering SMS is a further barrier. There are currently no dedicated agents for the implementation of SM policy, which is unlike the implementation of drug recommendations by doctors (SP, UK). Additionally, health professionals who could potentially offer SMS are not necessarily capable and qualified to do so with differences of training in community orientated approaches. In the UK, it is primarily practice nurses who deal with day to day diabetes care. In similarity to the Netherlands they are reported to have little training in dealing with the social and psychological consequences of living with a condition, which are key aspects of SMS.

> *I think one of the problems is that there isn't very much education and training for practice nurses and too often they are taught by their GP… Too often they're brought in and in reality all they're doing is ticking boxes for QOF (UK, 13)*

I think it would be wonderful if we had another model. Not like the healthy life centres, but a mastery centre with groups, physical activities and things like that. Like local learning and mastery courses for patients with DM2 with a great focus upon mastery. (NO, 13).

Where there has been adoption of SMS at the practice level it is seen as contingent on balancing conflicting priorities between a commitment to providing the best possible care to patients and maximising financial gain. An environment of incentives runs counter to lifestyle and behavioural interventions. Barriers to implementation are identified as lying outside the healthcare system and related to marketization imperatives. The impact in Norway of the self-employed status of primary care professionals was identified as making it difficult to regulate activity:

...there are challenges. Like we need paths for safe walking and bicycling to schools and so on – but who will argue this into the planning? The physicians are in their offices and they are educated to repair illness. It needs to be systematized cooperation. GPs need to be linked to the primary prevention as counselors, linked into groups - in community health services - working with primary prevention.. ….the GP scheme has many great benefits, but the communities need to have good medical advisors. …... The negative with GPs is that they are only concerned with treating patients. But someone must consider public health. And the municipality can do this.. (NO, 15)

…there's a suggestion in QOF that to achieve some of your diabetes points you are going to have to refer people to a structured education programme and at the moment there don't seem to be any structured educational programmes… Well there should be a double agreement with an outcome agreement but I think, actually I don't think we're very good at commissioning, we don't always hold people to the target (UK, 2)

A lack of sustainable funding contributed to making the availability of programmes a permanent feature of the healthcare environment. In NO, UK, NL narratives of SMS delivery through the healthcare system was characterised by concerns from respondent about an implementation gap.. The respondents viewed the age of austerity though undesirable in many respects as providing a potential to challenge vested interests and encourage cooperation. In the more poorly resourced countries of Bulgaria and Greece stakeholders identified a lack of resources and inadequate changes in the organisation and funding of the healthcare system as challenges shaping CIM. SMS availability was described as being non-existent or low down the priorities of policy makers and practitioners as a result of overriding concerns with the overall functioning and sustainability of the healthcare system and universal access to healthcare. A salient discussion point was the growing number of people who could not afford health insurance or had limited access to services and medications.

As a health care practitioner in primary care settings I can definitely say that I am not aware of any officially health policy for type 2 diabetes. There is no official guidance,

any training program or some other kind of support for diabetic patients in primary care ...Definitely there is none (GR, 7).

Healthcare institutions consider that the problem with the disease end with a 100% payment of medications for treatment, the rest is a patient's concern (BG, 15).

Gaps in healthcare provision were to some degree addressed through ad hoc measures. In GR accounts described an informal system of 'street clinics' where services to uninsured patients are provided by healthcare professionals who have been made redundant and medications accessed through social drugs banks. Within a context of weak state involvement and limited financial resources SMS is provided for by pharmaceutical companies.

The companies — producers of insulin – Novo Nordisk, LILLY and Sanofi invest mostly in training, whereas the companies who offer peroral therapy are dozens and very active in patients' training, but it is rather the companies that offer monitoring equipment and glucometers that should be involved in providing training for work with their equipment because the doctors cannot spare the time to explain such details to patients as diet and physical exercise (BG, 13).

They contribute to doctor/patient training because they create a number of nutritional programs, dietary guidelines, all of which are scientifically significant because they are made by nutritionists –people who have an understanding of these things, so they do help; also self-help material is available, which illustrates exactly what a patient needs to do, and it is explained easily, which helps (GR, 5).

Whilst respondents saw the positive role played by pharmaceutical companies none considered such arrangements a satisfactory alternative to state led provision. One respondent commented that the dominance of drugs companies in BG is so complete that: *"there is no patients' NGO in Bulgaria, which is not representative of a specific pharmaceutical company..." (BG, 10).* The negative impact that unregulated influence of drugs companies was recognised vis:

Patients with diabetes are frequently subject to strong and harsh therapy because the lighter ones are not paid. (for)…, this is tolerated by the healthcare system, whereas the companies interested in these clinical pathways instruct the doctors to direct their patients to treatment with their medications – thus there is a twisted consumption of medicines, healthcare services and public resources ….. Expensive medicines are being bought without therapeutic justification, we turn our patients into dependents on the system.. (BG, 8).

...they influence by increasing the number of diabetic medicines available for treatment to an extent greater than that which is required. In other words, they surely influence the prevention policy, and the policy of interventions from the health professionals. (GR, 7).

There was recognition by UK, NL, NO respondents that where interactions between health practitioners, policy makers and representatives of pharmaceutical companies is highly regulated the influence of pharmaceutical companies on policy and prescriptions is present but more circumscribed:

> *[…] They will sponsor websites, they will sponsor literature, they will sponsor things like that so it becomes a drip feed of people's awareness that there are these sort of different types of drugs available that might be better than what your GP already gives you and you should ask for them to use a different assertive type citizen type approach which we're not used to in this country but I think it might be something that happens (UK, 1).*

Discussion

The infrastructure and culture for supporting behavioural change and living well with a long term condition is driven to a significant extent by political decision-makers the socio-economic and policy environment and the ethos and delivery of CIM in health care systems. This study highlights the views and experiences of stakeholders and experts in six countries about a set of implicit influences operating in a socio-economic policy context likely to shape the perception and uptake of SMS at the level of individual action. Previous research has illuminated the presence of a powerful set of vested interests driving the commodification of long term condition management and the involvement of private business in public health programmes [18]. The socio-cultural and policy -influences and how these play out is relevant for understanding the limits of the current policy focus with its emphasis on individualised action as the bases of stepped changes to improved self-management. The analysis of stakeholder accounts point to the influences that need to be taken into account for a broader focus to become manifest in a new generation of SMS practices and policies

A cross-national perspective illuminated contrasts and commonalities with a generic under acknowledgement of the social environmental influences on diabetes SM . The latter included an absence of focus on inequalities, stigma, food company relationships relevant to over consumption and the inability of policy makers to regulate processes and environments relevant to chronic illness management. A bio-medical orientation to the shaping of incentives in chronic disease management was identified as a factor translating into SMS resources used by health care systems to encourage SMS. Narratives consistently referred to the *inter-relationship* between environmental facets and the shaping of and impact on the choices that people had to make at an individual action level. All partner countries identified problems with environments promoting diabetogenic diets. There was recognition of the need to address the nature of inequalities and the social cultural and structural barriers to the evaluation and implementation of approaches to CIM in European countries [19].

The latter points to a the relevance of a focus of exploring relationships between institutions responsible for policy making and SMS in terms of understanding the contradictions of modern welfare state and the‘ crises of crisis management' . The self management agenda within which the sub-system or space within which chronic illness is being managed represents an element of the activities related to the welfare state as an adaptive system of survival and constant state of contradiction in attempts to progress and protect the mass of the population in terms of health and well-being whilst being subject to the commodifying logic of capitalism and associated institutional logics[20,21]. Our analysis of the narratives of informants resonate with these notions in uncovering the tensions and interpretation of policy making which encourage dedicated policies for individual action to address a problem of the proliferation of the costs and morbidity associated with long term conditions through more effective self- management whilst needing to appease or incorporate other parties who look to modern welfare states to promote their interests . The findings point to the continuing disparities between states in the EU becoming manifest at the level of policies about self-management support. Initiatives have been variably introduced in European countries and inequalities and disparities in the relevance and scope of implementation exist between poorer and richer countries. Thus in poorer European settings a coherent overall policy about SMS is overshadowed by substantial inequalities and the failure to deal with the needs which are usually met in more affluent partner countries. Differences between settings are entwined with the contextual and cultural specificities of each setting in the articulation of SMS policy and practice. Specifically, our analysis pointed to the differential impact of poverty and austerity impacting on personal SMS environments. Bulgaria and Greece, and to a lesser extent Spain are characterised in relative terms to, the other three countries by poorer socio economic circumstances levels of austerity and a less adequately funded health care infrastructure . Differences were identified in relation to the existence and impact of dedicated policies on SM and the planning and delivery of resources and services orientated to CIM (see Table 3 above). In the more affluent countries it was what was going *wrong* with policies or in the organisation of delivery of services and resources associated with CDM that was emphasised rather than the absence of policies per se. Discussions about the latter made little sense where austerity and the lack of resources more generally predominated and the lack of or diminishing funding from the state might have accounted for the opening up of a space for commercial interests to play more of a role in commodifying chronic illness management. Drug companies were viewed as playing an explicit role reinforcing a focus on the bio medical with the potential to impact on the dynamics of chronic illness interactions and outcomes between practitioners and patients than better off countries where vested interests are managed indirectly through influence in the funding and orientation of research and development. Similarly, differences between affluent and less affluent settings were identified in the extent to which primary care services were seen to have introduced behavioural interventions [22] compared to the relegation of behavioural interventions to incentivised biomedical elements of practice . Finally there were variations in the extent to which the neo-liberal values of individual responsibility vied with taking precedence over and with the more collective and regulatory and redistribution capacities of the state .Some respondents noted policies that promoted exercise while not offering structural solutions to food consumptions and exercise environments whilst others had the necessary physical and social infrastructure (e.g. cycling-friendly environment).Respondents

from countries with well-established welfare states were more likely to report concerns with the growing tendency of opening up long term condition management to profit making whereas in others inadequacies in state capacity and the pressure on the state to prioritise the interests of business over other stakeholders were taken for granted Respondents in Greece, UK, and Norway reported concerns with the erosion of social rights and the need to confront the interests and power of pharmaceutical and food companies.

Limitations of the study

The types of stakeholders interviewed and thus to an extent the pursuance of similar topics to the same depth varied across countries and between respondents. However, this also reflected the differing influences at play and the extent to which SMS is a tangible and visible entity in the policy environment. Inevitably judgements about relevant processes were shaped by the values and foci of the respondents. Nonetheless, there was a significant degree of consistency in the key influences identified by respondents across and within partner countries. We followed a common analysis framework and group discussions in order to minimize methodological and interpretative differences across different national contexts.

Conclusion

SMS policy and practice might be designed with greater attention being paid to the limitations emanating from broader level influences on SMS. A key challenge lies in how to harness the power of influences that extend beyond the individual in bringing about changes to behaviour [22,23,24], and in confronting the vested economic and political interests that bloc the building of health-wise environments. Policy making, which has been influential in promoting the concept of SMS in some European countries, might be usefully extended to other in terms of informing the development of interventions which recognise elements of policy which operate at different levels and which impact on a setof conditions operating in a broader context

Competing Interests

The authors declare that they have no competing interests

Authors' contributions

AR and IV authored and are guarantors of this paper. AR,IV,AK,MW,CF,ET,ET,IF,MS,PR,CL made contributions to the conception and design, AR,IV,MJP,ET,MCP,CF,JK,NR,MS, ISRK,MW,PR CL were involved with the collection of data, analysis and interpretation of data and in drafting and revising the manuscript and have given final approval of the version to be published. This work has been funded by the Seventh Framework Programme of the European Commission (Grant agreement no: 279081 'EU-WISE').

Acknowledgements
We would like to acknowledge the work and contributions of EU-WISE researchers including Dr Sue Shea, Agapi Agelaki (University of Crete).

References

1. Escolar-Pujolar A, Bacigalupe A, San Sebastian M. European economic crisis and health inequities: research challenges in an uncertain scenario. *International Journal for Equity in Health* 2014, 13:59.
2. Grintsova O, Maier W, Mielck A. Inequalities in health care among patients with type 2 diabetes by individual socio-economic status (SES) and regional deprivation: a systematic literature review. *International Journal for Equity in Health* 2014, 13:43.
3. Vassilev I, Rogers A, Sanders C, Cheraghi- Sohi S, Blickem C, Brooks H, Kapadia D, Reeves D, Doran T, Kennedy A. Social status and living with a chronic illness: an exploration of assessment and meaning attributed to work and employment. *Chronic Illness* 2014, 10(4):273-90.
4.Rise MB, Pellerud A, Rygg LØ, Steinsbekk A. Making and maintaining lifestyle changes after participating in group based type 2 diabetes self-management educations: a qualitative study. PLoSONE. 2013; 8(5): e64009. doi:10.1371/journal.pone.0064009
5.Glazier RH, Bajcar J, Kennie NR, Willson K. A systematic review of interventions to improve diabetes care in socially disadvantaged populations. Diabetes Care 2006, 29(7):1675-1688.
6. Kousoulis AA, Patelarou E, Shea S, Foss C, Ruud Knutsen IA, Todorova E, Roukova P, Portillo MC, Pumar-Méndez MJ, Mujika A, Rogers A, Vassilev I, Serrano-Gil M, Lionis C. Diabetes self-management arrangements in Europe: a realist review to facilitate a project implemented in six countries. *BMC Health Services Research* 2014, 14:453.
7. Stuckler D, McKee M, Ebrahim S, Basu S. Manufacturing epidemics: the role of global producers in increased consumption of unhealthy commodities including processed foods, alcohol, and tobacco. *PLoS Med* 2012, 9: e1001235.
8. Macknbach JP, Karanikolos M, McKee M. The unequal health of Europeans: successes and failures of policies. *Lancet* 2013, 381: 1125-1134.
9. Vassilev I, Rogers A, Blickem C, Brooks H, Kapadia D, Kennedy A, Sanders C, Kirk S, Reeves D. Social networks, and the ‘work’ and work force of chronic illness self-management: A survey analysis of personal communities. *PLOSONE* 2013, 8: e59723.
10. Rogers A, Vassilev I, Kennedy A, Blickem C, Reeves D, Brooks H. Why less may be more?: A mixed methods study of the work and relatedness of 'weak' ties in supporting long term condition self- management. Implementation Science 2014, 9: 19.
11. Reeves D, Blickem C, Vassilev I, Brooks H, Kennedy A, Richardson, G, Rogers A. The Contribution of Social Networks to the Health and Self-Management of Patients with Long-Term Conditions: A Longitudinal Study. *PLOSONE* 2014, 9: e98340.
12. Bajekal M, Scholes S, Love H, Hawkins N, O'Flaherty M, Raine R, Capewell S. Analysing Recent Socioeconomic Trends in Coronary Heart Disease Mortality in England, 2000–2007: A Population Modelling Study. PLOS MEDICINE 2012, 9: e1001237.

13. Kennedy A, Bower P, Rogers A. Support for self care for patients with chronic disease. BMJ 2007, 335: 968.
14. Rogers A, Bury M, Kennedy A. Rationality, rhetoric and religiosity in health care: the case of England's Expert Patients Programme. International Journal of Health Services 2009, 39: 725-747.
15. Bower P, Blakeman T, Kennedy A, Protheroe J, Richardson G, Rogers A, Sanders C: What influences people to self care? *NPCRDC, The University of Manchester* 2007, http://www.population-health.manchester.ac.uk/primarycare/npcrdc-archive/Publications/WHAT_INFLUENCES_PEOPLE_TO_SELF_CARE_MARCH_2009.pdf. Accessed 7 May 2015.
16. Silverman D. Qualitative research: theory, method and practice. London: Sage; 1997.
17. Doran T, Kontopantelis E, Valderas J, Campbell S, Roland M, Salisbury C, Reeves D. Effect of financial incentives on incentivized and non-incentivized clinical activities. Evidence from the UK's Quality and Outcomes Framework. British Medical Journal. 2011, 342:10.1136/bmj.d3590
18.Greenhalgh T, Procter R, Wherton J, Sugarhood P Shaw S. The organising vision for telehealth and telecare:discourse analaysis. BMJ Open. 2012. **2**:e001574. doi:10.1136/bmjopen-2012-001574
19. Knai C, Nolte E, Brunn M, Elissen A, Conklin A, Pedersen JP, Brereton L, Erler A, Frølich A, Flamm M, Fullerton B, Jacobsen R, Krohn R, Saz-Parkinson Z, Vrijhoef B, Chevreul K, Durand-Zaleski I, Farsi F, Sarría-Santamera A, Soennichsen A. Reported barriers to evaluation in chronic care: experiences in six European countries. Health Policy. 2013, 110: 220-228.
20. Offe C and Lenhardt G. Social policy and the theory of the State. In Keane J editor. Contradictions of the Welfare State. London: Hutchinson; 1984.
21. Pilgrim D. The British welfare state and mental health problems: the continuing relevance of the work of Claus Offe. Sociology of Health and Illness. 2013, 34(7): 1070-84.
22. Lionis C, Symvoulakis E, Markaki A, Vardavas C, Papadakaki M, Daniilidou N, Souliotis K, Kyriopoulos I. Integrated primary health care in Greece, a missing issue in the current health policy agenda: a systematic review. Int J Integr Care. 2009, 9: e88.
23. European Commission. Patient involvement: aggregate report. Eurobarometer qualitative study. 2012, http://ec.europa.eu/public_opinion/archives/quali/ql_5937_patient_en.pdf Accessed 7 May 2015.
24. Koetsenruijter J, van Lieshout J, Vassilev I, Portillo MC, Serrano M, Knutsen I, Roukova P, Lionis C, Todorova E, Foss C, Rogers A, Wensing M. Social support systems as determinants of self-management and quality of life of people with diabetes across Europe: study protocol for an observational study. Health and Quality of Life Outcomes. 2014, 12: 29.

Table 1: Background of key informants*[iii]

	Professionals			Policymakers	Academics	Managers/representatives of Drug Companies or |Health Units
	Physician[3]	Nurse	Other			
Bulgaria	11	1	3	5	5	5
Greece	6	2	7	3	3	3
Netherlands	2	2	11	6	3	1
Norway	5	4	6	7	2	2
Spain	5	3	7	6	7	2
UK	6	1	8	3	9	3

Table 2: Key themes and sub-themes from respondent narratives

	BG	GR	SP	UK	NO	NL
**Social environmental influences on diabetes self-management**	+	+	+	+	+	+
Social inequalities impact on resources for SM				+	+	+
Public stigma and impact of portrayal of behavioural dispositions	+	+		+	+	
Media and the portrayal of stigma	+	+	+	+	+	

[3] The category "physician" states the level of education in medicine.

Diabetogenic food environment	+	+		+	+	
**Inability of policy to regulate processes and environments related to chronic illness management**	+	+	+	+	+	+
Lack of governments capacity to regulate the food supply chain	+			+	+	
Growing responsibility of localities stakeholders in a context of financial uncertainty			+	+	+	+
Challenges associated with the coordination, funding, and implementation of local commissioning of services				+	+	+
Extending the scope of voluntary and community groups and private provider involvement in SMS				+	+	
Few welfare resources and impact of austerity on local supply and demand for SMS	+	+		+		
**Bio-medical tendencies and incentives in primary care**	+	+	+	+	+	+
Gap between SMS policy and implementation within health services	+	+	+	+	+	
Inconsistent support for shift in healthcare provision towards better SMS		+	+	+		
Prevention/ public health interventions have a role in SMS	+	+	+	+	+	+
Insufficient policy level commitment to implementing SMS policies	+	+	+	+	+	
Lack of incentives for SMS		+		+		
Insufficient SMS tools and infrastructure in the health service	+	+	+	+	+	+
Drugs companies interests as barrier to implementing SMS	+	+	+	+	+	
Professionals interests as barrier to implementing SMS	+		+	+		
Growing involvement of patient groups			+		+	+
Financial crisis as an opportunity for changes in the healthcare system		+	+	+		
System level crisis as a dominant policy concern	+	+				
Drug companies providing SMS in the absence of state capacity	+	+	+			

TABLE 3: Key theme similarities and differences between partner countries

	Low and medium income countries			High income countries		
	BG	GR	SP	UK	NO	NL
Adequately funded health care infrastructure				++	++	++
Existence and impact of dedicated SM policies, services, and resources			+	++	++	++
Emphasis on nuances in SMS policies, resources, and delivery		+	+	++	++	++
Absence of dedicated and enacted policies related to inadequate provision, austerity, and lack of resources	++	++	+			
Drug companies viewed in terms of playing an explicit role in SM	++	++	++			
Drug companies in strong position to reinforcing biomedical focus in SM	++	++	++			
Focus on promoting SM at an individual level				++	+	++
Behavioural interventions introduced by primary care services				++	++	++
Presence of cycling-friendly physical and social environment					++	++
Expressed concerns with social rights & socialising healthcare cost		++		++	++	

'++' the statement is fully valid for this country; '+' the statement is only to some extent valid for this country

Please note that each country is assessed in relation to how all other countries in the sample, e.g. regional differences exist within all countries in the sample, but they are relatively small compared to those in Spain, partly due to their autonomous status.

---

[i] *The interviews were analysed using an agreed framework and relevant sections were translated into English.*

Acknowledgements
We would like to acknowledge the work and contributions of EU-WISE researchers including Dr Sue Shea, Agapi Agelaki (University of Crete),

[ii]

[iii] because some of our respondents could be described under different categories the overall count is in some cases over 15
